# Characterization of hemolymph phenoloxidase activity in two *Biomphalaria* snail species and impact of *Schistosoma mansoni* infection

**DOI:** 10.1186/s13071-016-1319-6

**Published:** 2016-01-22

**Authors:** Winka Le Clec’h, Timothy J. C. Anderson, Frédéric D. Chevalier

**Affiliations:** Department of Genetics, Texas Biomedical Research Institute, P.O. Box 760549, San Antonio, TX 78245 USA

**Keywords:** *Biomphalaria*, Phenoloxidase activity, Laccase, Hemolymph, Spectrophotometric assay, *Schistosoma mansoni*

## Abstract

**Background:**

*Biomphalaria* snails are the intermediate host of the blood fluke *Schistosoma mansoni*, which infect more than 67 million people in tropical areas. Phenoloxidase enzymes (POs), including tyrosinases, catecholases, and laccases, are known to play a role in the immune defenses of arthropods, but the PO activity present in *Biomphalaria spp*. hemolymph has not been characterized. This study was designed to characterize substrate specificity and reaction optima of PO activity in *Biomphalaria spp.* hemolymph as a starting point to understand the role of this important invertebrate enzyme activity in snail biology and snail-schistosome interactions.

**Methods:**

We used spectrophotometric assays with 3 specific substrates (L-tyrosine for tyrosinase, L-DOPA for catecholase, and PPD for laccase) and diethylthiocarbarmate (DETC) as specific PO inhibitor to characterize PO activity in the hemolymph of uninfected snails from two *Biomphalaria* species, and to determine the impact of the parasite *Schistosoma mansoni* on the PO activity of its *B. glabrata* vector.

**Results:**

We identified laccase activity in hemolymph from uninfected *B. glabrata* and *B. alexandrina.* For both species, the activity was optimal at 45 °C and pH 8.5, and located in the plasma. The *K*_*m*_ and *V*_*max*_ of PO enzymes are 1.45 mM and 0.024 OD.min^-1^ for *B. glabrata,* and 1.19 mM and 0.025 OD.min^-1^ for *B. alexandrina*. When the snail vector is parasitized by *S. mansoni*, we observed a sharp reduction in laccase activity seven weeks after snail infection.

**Conclusions:**

We employed a highly specific spectrophotometric assay using PPD substrate which allows accurate measurement of laccase activity in *Biomphalaria spp.* hemolymph. We also demonstrated a strong impact of the parasite *S. mansoni* on laccase activity in the snail host.

**Electronic supplementary material:**

The online version of this article (doi:10.1186/s13071-016-1319-6) contains supplementary material, which is available to authorized users.

## Background

Aquatic snails of the *Biomphalaria* genus are the intermediate hosts of the blood fluke *Schistosoma mansoni* [1, 2], trematodes that infect 67 million people in Africa and South America [3–5]. When parasite eggs are expelled with human faeces in water, miracidia larvae hatch and actively search for its snail vector. Larvae penetrate the snail head-foot, differentiate into primary sporocysts and then asexually proliferate to generate secondary sporocysts. After approximately a month of infection, secondary sporocysts release the first cercariae, the human infective larval stage of the parasite, through the body of the snail, into the water. The *Biomphalaria* immune response is mounted both by cellular effectors (*i.e.* via the hemocytes, [6–10]) and humoral factors (for example FREPs [11, 12], SOD1 [13], Biomphalasin β-PFT [14], and lectins [15]). Many snail humoral factors have been carefully characterized in the *Biomphalaria* genus to identify the resistance mechanisms to schistosome infection. However no clear characterization of the phenoloxidase (PO) activity of the *Biomphalaria* snail hemolymph was attempted. This is surprising because PO activity is considered to be an important component of the humoral response [16] and an immunocompetence parameter in many arthropods [17–19]. The central aim of this work is to characterize the specific PO activity present in *Biomphalaria* hemolymph: this is an essential prerequisite for studies aiming to understand the role of PO activity in snail biology.

PO enzymes play a key role in wound healing [16], tissue pigmentation [20, 21], and reproductive process [22–25]. They are also involved in innate immune defense against intruding pathogens, being the last component of a reaction cascade called the “proPO activating system” [16, 26]. This cascade is triggered when pathogen molecules are detected and stimulate the activation of proPO enzymes into PO enzymes through the action of serine proteases. The active POs then convert phenolic or amine compounds in dopachrome and then melanin, which have cytotoxic activities damaging pathogen cells [16].

PO enzymes are copper-containing enzymes [27] and fall into three groups defined by their substrate specificity: (i) tyrosinases catalyze hydroxylation of monophenols and oxidation of *o*-diphenols, (ii) catecholases oxidize *o*-diphenols and (iii) laccases oxidize *o*-diphenols, *p*-diphenol and *p*-diamines [28]. Invertebrates possess the 3 different PO activities in various tissues [29–31], unlike vertebrates where only the tyrosinase activity is present [32]. Invertebrate PO activity can be easily measured and characterized in vitro using specific substrates. Assays conducted in the presence of exogenous serine proteases (like trypsin enzymes) measure all the PO activity present in an individual (total PO activity), while assays conducted in the absence of exogenous serine proteases measure the PO activity that can be activated during infection (intrinsic PO activity). Among all the substrates used to measure PO activity, L-DOPA (*o*-diphenol) is the most frequently used because it is non-specific and therefore does not require any prior knowledge about the enzyme involved in PO activity [28]. However, this substrate has multiple disadvantages because (i) its non-specific nature may lead to low oxidation efficiency and inaccurate measurement [28], (ii) it can be metabolized by other enzymes such as peroxidases [33, 34] and (iii) is highly susceptible to auto-oxidation [35].

Several studies have examined PO activity in pulmonate snails, but importantly all of these studies used L-DOPA, rather than specific substrates, and have therefore provided ambiguous results. For example, one study on *Lymnaea stagnalis* [36] suggests that peroxidase activity rather than PO activity is responsible for L-DOPA oxidation but suffers from methodological limitations, because PO and peroxidase activity cannot be distinguished. In this study the failure to detect PO inhibition may be due to (i) the inefficacy of the unique PO inhibitor used (phenylthiourea) and (ii) an unusually short time to dopachrome formation measurement for invertebrate hemolymph (5 min). In another study on *Lymnaea*, Seppälä and Leicht [37] quantified PO-like activity but did not use a specific inhibitor to verify that the activity observed was due to oxidation of L-DOPA by PO enzymes. A study on *B. glabrata* snails [38] quantified PO activity in hemocytes six hours after adding L-DOPA, but without an L-DOPA auto-oxidation control, making it difficult to conclude true PO activity rather than substrate auto-oxidation. As a consequence, specific PO activity (tyrosinase, catecholase or laccase) remains poorly understood in snail hemolymph, and studies using specific substrates are required. Moreover, while infection with *S. mansoni* intramolluscan stages has a negative impact on the tyrosinase activity in the *B. glabrata* albumen gland [39], nothing is known about the effects of the parasite on the PO activity in the snail hemolymph, a tissue in intimate contact with *S. mansoni* larvae.

To fill this knowledge gap, we characterized PO activity in the hemolymph of uninfected *Biomphalaria spp.* (*B. glabrata* and *B. alexandrina*) using three different specific substrates (L-tyrosine, L-DOPA and *p*-phenylenediamine (PPD)) and employed a specific and accurate assay to measure this activity. We then determined general characteristics of PO activity, including the temperature and pH optima, the exact location of action, Michaelis-Menten constant (*K*_*m*_) and maximum velocity (*V*_*max*_). Finally, we measured the impact of *S. mansoni* infection on PO activity of the *B. glabrata*’s hemolymph over the pre-patent period (during 4 weeks after the exposure to the parasite, when primary sporocysts grow and produce several generations of secondary sporocysts in the snail tissues) and 5 weeks over the patent period (when secondary sporocysts produce cercariae released from the snail).

## Methods

### Ethics statement

This study was performed in strict accordance with the recommendations in the Guide for the Care and Use of Laboratory Animals of the National Institutes of Health. The protocol was approved by the Institutional Animal Care and Use Committee of Texas Biomedical Research Institute (permit number: 1419-MA-0).

### Maintenance of snails *Biomphalaria glabrata* and *alexandrina*

We characterized phenoloxidase (PO) activity in the hemolymph of two different *Biomphalaria* species, both intermediate hosts of *Schistosoma mansoni*. We used 385 inbred albino *B. glabrata* (line Bg26 derived from 13-16-R1 line [40]) and 185 outbred pigmented *Biomphalaria alexandrina* (from Theodor Bilharz Research Institute, Egypt) in the experiments presented.

Uninfected snails were reared in 10-gallon aquaria containing aerated freshwater at 26-28 °C on a 12 L-12D photocycle and fed ad libitum on green leaf lettuce. All snails used in this study had a shell diameter between 10-14 mm.

### Hemolymph collection on *Biomphalaria spp.* snails

We collected hemolymph immediately before assaying PO activity. An advantage of *Biomphalaria* snails is their large size and the flat shape of their shell allowing an easy access to the heart and the collection of ~100 μL of hemolymph. For each individual snail, we disinfected the shell with 70 % ethanol, and we collected total hemolymph by heart puncture using a 1 mL syringe and a 22 gauge needle. The hemolymph collected was immediately placed in a 1.5 mL microtube on ice.

### Characterization of phenoloxidase activity in the hemolymph of *Biomphalaria spp.*

Three specific substrates for PO enzymes were tested: (i) L-tyrosine (monophenol; Sigma) metabolized by tyrosinase only, (ii) L-DOPA (*o*-diphenol; Sigma) metabolized by catecholase, tyrosinase and laccase enzymes, and (iii) *p*-phenylenediamine (PPD; *p*-diamine; Sigma) only metabolized by laccase enzyme [41, 42].

We detected and characterized PO activity in the hemolymph of *Biomphalara spp*. by measuring the optical density (OD) of the colour reaction product (*i.e.* dopachrome) formed by the oxidation of each substrate. The OD was measured using a spectrophotometer at λ = 465 nm when using PPD (according to maximum absorption of the product of PPD oxidation by the PO enzyme, Additional file 1: Figure S1) and at λ = 490 nm when using L-tyrosine and L-DOPA [43].

In each sample test well of a 96-well optical plate (Corning), we added 10 μL of hemolymph to 40 μL of cacodylate buffer (10 mM sodium cacodylate (Sigma) and 10 mM calcium chloride (Sigma) in distilled water, pH = 8.4). Each sample test was coupled to a control test where 10 μL of the same hemolymph sample was added to 40 μL of 10 mM diethylthiocarbamate (DETC (Sigma) in cacodylate buffer, pH = 8.4). DETC is known to be a specific inhibitor of PO enzymes [38, 39, 44]. A substrate auto-oxidation control was also performed, where the hemolymph sample was replaced by 10 μL of distilled water. The values obtained for this control were automatically subtracted from the test and control wells values for each experiment.

In order to measure the total PO activity in the hemolymph, we added an exogenous protease (40 μL of trypsin prepared at 1 mg.mL^-1^ in distilled water) mimicking the action of the serine protease of the PO cascade to each well (test and control). Following the addition of trypsin, the assay was incubated 45 min at 37 °C (the optimal temperature for trypsin protease). Fresh substrates (L-tyrosine, L-DOPA and PPD) were prepared at 10 mM in cacodylate buffer ten minutes before usage and 120 μL of substrate were added to the wells followed immediately by a plate reading. We tested each sample with the 3 substrates. Dopachrome formation was spectrophotometrically monitored every 15 min for 6 h at 37 °C and λ = 465 nm and λ = 490 nm, using a SpectraMax M1 (Molecular Devices). Comparisons between substrates were done using absorbance values obtained 4 h after adding the substrates at 10 mM, before reaching the plateau phase of the PO activity.

### Influence of the temperature and pH on the laccase activity in the hemolymph of *Biomphalaria spp*.

To assess the effect of temperature on laccase activity in the hemolymph of *Biomphalaria spp*., we conducted laccase assays as described above (using 50 mM PPD, substrate in large excess). We determined dopachrome formation every 15 min for 6 h using a temperature range from 30 to 60 °C at λ = 465 nm.

We also examined the effect of pH on laccase activity. We used the same assays to those described but using cacodylate buffers with a pH ranging from 4.5 to 12.5 and spectrophotometric monitoring (every 15 min for 6 h) at 45 °C (optimum temperature) and λ = 465 nm.

### Determination of the Michaelis constant (*K*_*m*_) and the maximum velocity (*V*_*max*_) of the laccase-like enzyme from the hemolymph

To determine the Michaelis constant (*K*_*m*_) and the maximum velocity (*V*_*max*_) of the laccase-like enzyme in the *Biomphalaria* hemolymph, we conducted spectrophotometric assays as described above using a final PPD concentration range from 0.891 mM to 14.28 mM. We monitored the enzymatic kinetics for both snail species during every 15 min for 6 h at 45 °C (optimal temperature) and λ = 465 nm. *K*_*m*_ and *V*_*max*_ were obtained from the Hanes-Woolf equation, a linear transformation of the Michaelis-Menten equation [45] using the formula:

$$ \frac{\left[S\right]}{V}=\frac{\left[S\right]}{V_{max}}+\frac{K_m}{V_{max}} $$

where [S] is the substrate concentration in mM, V and *V*_*max*_ are the velocity and the maximum velocity in OD.min^-1^ respectively, and *K*_*m*_ the Michaelis constant in mM.

### Location of the laccase activity in the hemolymph of *Biomphalaria spp.*

Hemolymph collected from the snail comprises two fractions: the humoral fraction (plasma) and the cellular fraction containing the immune cells (hemocytes). Laccase activity was determined and characterized in the complete hemolymph, as well as in plasma and hemocytes. We centrifuged the samples at 300 × *g* (at 4 °C for 5 min) to separate hemolymph into the two fractions. To be sure that the hemocyte fraction did not contain plasma, the cells were washed 2 times (300 × *g* at 4 °C for 5 min) in PBS (pH 8.5) then resuspended in the same volume of PBS. We checked for the presence of cells in the hemocyte fraction as well as the integrity of the cells (mean viability of the hemocytes ± sd: 87 ± 8 %) using an automatic cell counter (BioRad) (10 μL of the cellular fraction combined with 30 μL of trypan blue 0.4 % (Sigma)). We used the same approach to check for the absence of hemocytes in the plasma fraction.

We conducted spectrophotometric assays both with trypsin (total laccase activity) and without trypsin (intrinsic laccase activity), using whole hemolymph and the two fractions (plasma and hemocyte). Note that our assay for laccase activity uses 37 °C rather than optimal 45 °C because trypsin activity is optimal at 37 °C and because the spectrophotometer works efficiently at this temperature, and 37 °C allows comparisons with previous PO activity studies.

### Impact of *S. mansoni* on *B. glabrata* laccase activity in the hemolymph, across the infection

To assess the impact of the *S. mansoni* parasite to its intermediate host *B. glabrata,* we infected 100 snails with *S. mansoni* parasites from SmLE population. A control experiment was performed at the same time, using 100 uninfected snails. The SmLE schistosome population was originally isolated from a patient in 1965 in Belo Horizonte (Minas Gerais, Brazil), and has since been maintained in the laboratory [46], using NMRI line *B. glabrata* as intermediate host and syrian golden hamster (*Mesocricetus auratus*) as definitive hosts. Miracidia were hatched from eggs recovered from 45-day-infected hamster livers. The livers were homogenized and the eggs were filtered, washed with normal saline (154 mM calcium chloride (Sigma), pH 7.5), transferred to a beaker containing freshwater, and exposed to artificial light to induce hatching. Snails were individually exposed to 10 miracidia (to maximize the number of infected snails) then maintained in trays for 9 weeks. We covered trays with a black plexiglass lid after 3 weeks to reduce cercarial shedding. Four weeks post-exposure and then once a week, we exposed infected snails to artificial light to induce cercarial shedding.

We sampled 10 infected and 10 control (uninfected) snails per week for hemolymph collection and assessment of infection status. We collected hemolymph from both infected and uninfected snails as described above (see Hemolymph collection on *Biomphalaria spp*. snails section)*.* The total and intrinsic laccase activity (with and without trypsin protease, respectively) was then spectrophotometrically monitored as described. Time-lapse series were built using absorbance values obtained 2 h after adding the PPD substrate, before reaching the plateau phase of the laccase activity.

During the pre-patent period (*i.e.* the first four weeks before the first cercarial shedding), we assessed the infection status of each snail after hemolymph collection by fixing each snail in Railey-Henry solution [47] and then dissecting them to observe the presence of primary sporocysts in the head-foot region, and secondary sporocysts in the hepatopancreas.

### Statistical analyses

All statistical analyses and graphs were performed using R software (version 3.0.1). When data distribution did not follow a normal distribution (Shapiro test, p < 0.05), results were compared with a Kruskal-Wallis followed by Dunn’s multiple comparison test or simple pairwise comparison (Wilcoxon-Mann-Whitney test). When data were normally distributed, results were compared with an ANOVA followed by Tukey’s multiple comparison test or simple pairwise comparison *t*-test.

## Results

### Laccase activity characterized in the hemolymph of *Biomphalaria spp.*

**Fig. 1:**
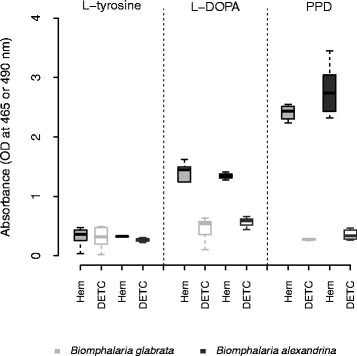
Characterization of PO activity in the hemolymph of *B. glabrata* (n = 15) and *B. alexandrina* (n = 15). PO activity in the hemolymph of both species of snails were assessed with three PO substrates: L-tyrosine (metabolized by tyrosinase only), L-DOPA (metabolized by catecholase, laccase and tyrosinase) and *p*-phenylenediamine (PPD) (metabolized by laccase only). No PO activity was detected with L-tyrosine whereas strong activity was measured with PPD, demonstrating the presence of laccase activity in the hemolymph. Significant inhibition of the activity with diethylthiocarbamate (DETC), a specific inhibitor of PO enzymes, demonstrates the specificity of the activity measured. Comparisons between substrates were done using absorbance values obtained 4 h after adding the substrates at 10 mM, before reaching the plateau phase of the PO activity

We found the strongest phenoloxidase (PO) activity (*i.e.* dopachrome synthesis) in the hemolymph of both *B. glabrata* and *B. alexandrina* 4 h after adding the *p*-phenylenediamine (PPD) substrate, which is the specific substrate of laccase enzymes (Fig. 1). Test wells (containing hemolymph) and the inhibition control wells (containing hemolymph + diethylthiocarbamate (DETC) the specific competitive inhibitor of PO enzymes) showed a strong difference for both *B. glabrata* (*t*-test, *p* < 1.10^-10^) and *B. alexandrina* (*t*-test, *p* < 0.0001), demonstrating that no other enzymes (such as peroxidases) were involved in this reaction.

We detected PO activity after 4 h using the L-DOPA substrate, which can be metabolized by all three types of enzyme, on the same hemolymph samples. Test wells and inhibition control well showed a strong difference for both *B. glabrata* and *B. alexandrina* (*t*-test, *p* < 0.0001 and *p* = 0.004 respectively; Fig. 1). We detected no PO activity when using L-tyrosine as substrate, showing that tyrosinase enzyme is not present in hemolymph of *Biomphalaria spp*. (Fig. 1).

For the two snail species, PO activity measured using PPD substrate is significantly higher than the activity assessed with L-DOPA (for *B. glabrata*, *t*-test, *p* < 0.0001 and for *B. alexandrina*, *t*-test, *p* < 0.0001). This demonstrates that a laccase-like enzyme is responsible for the PO activity measured in *Biomphalaria spp*. hemolymph.

### General characteristics of the hemolymph laccase activity: temperature and pH optimum, Michaelis constant (*K*_*m*_) and maximum velocity (*V*_*max*_) of *Biomphalaria spp.* enzyme

**Fig. 2:**
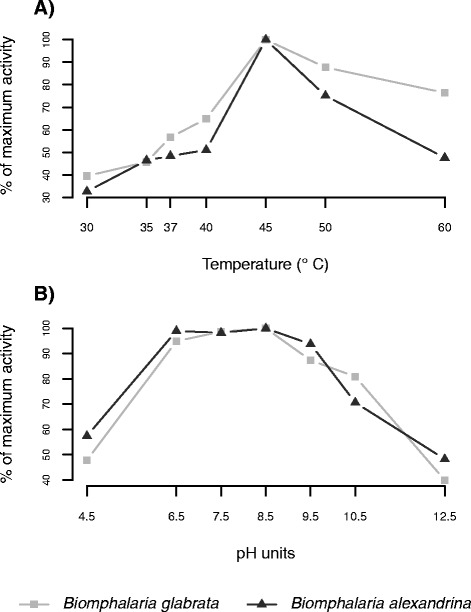
The effect of temperature and pH on the laccase activity in the hemolymph of *B. glabrata* (n = 70) and *B. alexandrina* (n = 70). Activity was measured as the amount of dopachrome formed from the oxidation of PPD in the hemolymph of both species, at the stated temperatures (**a**) and pH (**b**). Values are expressed as a percentage of the maximum activity, obtained at a 45 °C (**a**) and pH 8.5 (**b**)

We determined optimal parameters for the laccase-like enzyme from the hemolymph over a temperature range from 30 °C to 60 °C and a pH range from 4.5 to 12.5 (adjusted pH of the cacodylate buffer). The maximum activity of the hemolymph laccase-like enzyme is obtained at 45 °C for both snail species (Fig. 2a). Moreover, laccase activity was high from pH 6.5-8.5, but was the greatest at a pH of 8.5 (Fig. 2b), the physiological pH of *Biomphalaria spp.* hemolymph [38].

These parameters (temperature of 45 °C and pH of 8.5) were used to determine the Michaelis constant (*K*_*m*_) and the maximum velocity (*V*_*max*_) of the laccase-like enzyme for both snail species, for PPD concentrations ranging from 0.891 mM to 14.28 mM. Using the Hanes-Woolf equation, a *K*_*m*_ of 1.45 mM and a *V*_*max*_ of 0.024 OD.min^-1^ were calculated for the laccase-like enzyme of *B. glabrata* hemolymph, and a *K*_*m*_ of 1.19 mM and a *V*_*max*_ of 0.025 OD.min^-1^ were determined for the enzyme of the *B. alexandrina* hemolymph. These enzyme parameters showed no difference between the two snail species (*χ*^2^ test, *p* = 0.99 and *p* = 0.87 for *V*_*max*_ and *K*_*m*_ respectively).

### Laccase activity is located in the plasma of *Biomphalaria spp.* and enhanced by trypsin

**Fig. 3:**
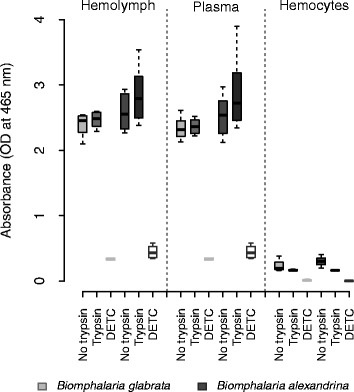
Location of the laccase activity inside the hemolymph of *B. glabrata* (n = 15) and *B. alexandrina* (n = 15). PO activity was measured in (i) the hemolymph, (ii) the acellular compartment (*i.e.* plasma) and (iii) the cellular compartment (*i.e.* hemocytes) of both snail species, with and without trypsin as protease activator. Diethylthiocarbamate (DETC) was used as inhibition control. Activity was measured as the amount of dopachrome formed from the oxidation of PPD substrate (10 mM), 4 h after the substrate addition. No difference in laccase activity was found between hemolymph and plasma for both species, and minimal activity was detected in the hemocytes

We measured laccase activity in the hemolymph, plasma and hemocytes from individual snails of the two species. Four hours after adding the PPD substrate, laccase activity was the same in the hemolymph and the plasma fraction for both *B. glabrata* and *B. alexandrina*. This was consistent with or without trypsin (Fig. 3). We detected laccase activity in the hemocytes for both snail species (*B. glabrata*, *t*-test, *p* = 0.0077; *B. alexandrina*, *t*-test, *p* = 0.0001; Fig. 3) but this was very low compared to the hemolymph or plasma (~10 fold less; Kruskal-Wallis test, *p* = 1.3.10^-9^). These results demonstrated that the vast majority of laccase activity is located in the humoral component of the snail hemolymph; the laccase-like enzyme is circulating in the plasma.

### Influence of parasite infection on laccase activity in the hemolymph of *B. glabrata*

**Fig. 4:**
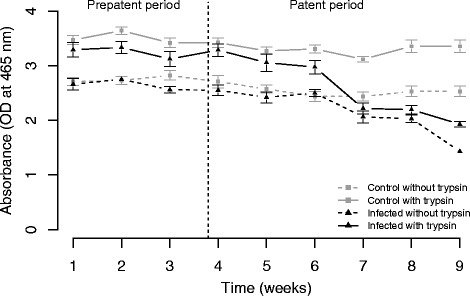
Impact of *S. mansoni* infection on laccase activity in the hemolymph of *B. glabrata*, across the infection (9 weeks). PO activity was measured as the amount of dopachrome formed from the oxidation of PPD substrate (50 mM), with and without trypsin as protease activator, 2 h after the substrate addition. Total (with trypsin activation) and intrinsic (without trypsin activation) laccase activity are shown in solid and dotted lines respectively. Grey lines refer to uninfected control snails and black lines to infected snails. *S. mansoni* has a strong impact on snail laccase activity (both total and intrinsic). This effect was not immediate but appeared after 6 weeks of infection

To investigate the impact of parasitism on *B. glabrata* phenoloxidase activity we infected *B. glabrata* with *S. mansoni* (SmLE) parasites and monitored PO activity over 9 weeks of infection (Fig. 4).

Overall, we observed a strong negative impact of *S. mansoni* infection on both total laccase (*i.e.* with trypsin, Kruskal-Wallis test, *p* = 1.03.10^-10^) and intrinsic laccase activity (*i.e.* without trypsin, Kruskal-Wallis test, *p* = 4.321.10^-7^). However, the impact of infection on hemolymph laccase activity is not immediate. During the first six weeks we observed minimal differences between the uninfected control and infected snails with trypsin (Welsh *t*-test, *p* = 0.048) and no differences without trypsin. However, we observed very strong differences from week 7 (with trypsin: Welsh *t*-test, *p* < 0.0001 for week 7 to 9 and without trypsin: Welsh *t*-test, *p* = 0.0233 for week 7, *p* = 0.0020 for week 8 and *p* < 0.0001 for week 9). The differences in laccase activity between infected and uninfected snails results from a strong reduction in laccase activity over time after week 6 in the infected snails both with and without trypsin (Kruskal-Wallis test followed by a Dunn’s post-hoc test, with trypsin: *p* = 1.187.10^-10^; without trypsin: *p* = 5.280.10^-11^). In comparison, we observed no change in laccase activity over time in the uninfected control snails with trypsin, and a slight change without trypsin (Kruskal-Wallis test followed by a Dunn’s post-hoc test, *p* = 0.01).

We observed a global increase in the PO activity after trypsin treatment in both infected (Kruskal-Wallis test, *p* = 6.416.10^-6^) and control snails (Kruskal-Wallis test, *p* < 0.0001). This result demonstrates the role played by serine protease in activation of the laccase-like enzyme in *B. glabrata*. However in infected snails, this difference is only seen during weeks 1 to 6 (Welsh *t*-test, *p* = 0.0093). From weeks 7 to 9, there is no difference, suggesting that no extra proPO enzyme can be activated.

## Discussion

In this study, we demonstrate that the PO activity from both *B. glabrata* and *B. alexandrina* hemolymph is laccase activity, and is located in the plasma. We then examined the impact of *S. mansoni* infection on the PO activity of *B. glabrata,* demonstrating a dramatic reduction from week 7 post-infection.

### Characterization of the phenoloxidase activity

We characterized specific PO activity in both *B. glabrata and B. alexandrina* using PPD substrate and inhibition using DETC. PPD substrate is specific to laccase and laccase-like enzymes, and has been previously used to demonstrate laccase activity in the hemolymph of the oyster *Crassostrea gigas* [43], the clam *Venerupis philippinarum* [48] and the abalone *Haliotis tuberculata* [49]. We found no formation of dopachrome with the L-tyrosine substrate, as seen in oyster [43] and abalone [49]. Some PO activity was measured with the L-DOPA substrate but this was significantly lower than with PPD. This result is most likely explained by the non-specific nature of the L-DOPA substrate: this is probably metabolized by the laccase-like enzyme but at a lower rate than the PPD. Hemocyanin, another copper-containing protein present in hemolymph that is known to show catecholase activity in crustaceans [50], does not show any phenoloxidase activity in *B. glabrata* [51] and therefore cannot be responsible for L-DOPA oxidation. PO inhibition by DETC, which chelates copper present in the enzyme [39, 44, 52, 53], confirms that the enzyme responsible for the metabolization of the PPD and the L-DOPA is a copper-containing metalloenzyme [28]. Our results illustrate the importance of specific characterization of PO activity, because this allows development of assays with much greater sensitivity as shown by the higher detection of activity with PPD than with L-DOPA. Specificity is also improved using PPD substrate and DETC inhibitor, as L-DOPA can be oxidized by peroxidase or can auto-oxidize [33, 35] resulting in false positive results for PO.

To further characterize PO activity, we determined reaction optima. The *Biomphalaria* laccase-like enzyme has maximum activity between pH 6.5 to 8.5 (with a maximum at 8.5) and at 45 °C which is consistent with the values reported for other mollusks (Table 1). The explanation for the high optimal temperatures found in many invertebrates is not yet known. One hypothesis is that PO temperature optima may be selected to correspond to the temperature generated by local inflammation at the wound site. Such temperature matching would increase the efficiency of the enzyme reaction while at the same time limit unwanted PO-related tissue damage elsewhere in the organism. These optimal parameters allowed determination of the *K*_*m*_ and *V*_*max*_ of the laccase-like enzyme for both snail species. *K*_*m*_ and *V*_*max*_ values were similar between *B. glabrata* and *B. alexandrina* but lower than other laccase-like enzyme characterized in gastropods and bivalves (Table 1), which reveals a higher affinity for the PPD substrate in *Biomphalaria spp*.

**Table 1:** Summary of the general characteristics of PO enzymes determined from hemolymph of several mollusks

Class	Species	Substrate used for PO assays	PO optimum temperature (°C)	PO optimum pH	Michaelis constant (*K*_*m*_) of PO enzyme identified (mM)^a^	*V*_*max*_ of PO enzyme identified (OD.min^-1^)^a^	References
Gastropods	*Biomphalaria glabrata*	PPD	45	6.5-8.5 Max:8.5	1.45	0.024	Present study
	*Biomphalaria alexandrina*	PPD	45	6.5-8.5 Max:8.5	1.19	0.025	Present study
	*Halitotis tuberculata*	PPD	ND	8.2	13.5	0.029	Le Bris *et al.*, 2014 49]
Bivalves	*Saccostrea glomerata*	L-DOPA	37	8	NA	NA	Aladaileh *et al.*, 2007 [64]
	*Crassostrae virginica*	L-DOPA	ND	6-7.5	NA	NA	Jordan and Deaton, 2005 [44]
	*Crassostera gigas*	PPD	ND	ND	45	0.00059	Luna-acosta *et al.*, 2011 [58]
	*Ruditapes philippinarum*	L-DOPA	40	7	NA	NA	Cong *et al.*, 2005 [53
	*Venerepis philippinarum*	PPD	40	8.4	14.46	0.23	Le Bris *et al.*, 2013 [48]
	*Chlamys farreri*	L-DOPA	45	6	NA	NA	Sun and Li, 1999 [65]

*ND* Not determined

*NA* Not applicable

^a^Michaelis constant (*K*_*m*_) and maximum velocity (*V*_*max*_) of PO enzyme are strongly dependent to the substrate used. We mentioned *K*_*m*_ and *V*_*max*_ values when they are assessed using PPD substrate only, as in the present study

We localized laccase activity in the plasma (*i.e.* acellular fraction of the hemolymph) in *Biomphalaria spp.* as observed in some other mollusks [48, 54, 55]. In comparison, Bahgat *et al.* [38] reported PO activity in hemocyte lysates, although no data was shown in their manuscript, and PO activity was tested in neither whole hemolymph nor plasma. Furthermore, their PO measurements were performed with the non-specific L-DOPA substrate 6 h after adding the substrate, at 405 nm (a suboptimal wavelength to quantify dopachrome formation which has a maximum of absorbance at 490 nm) and with no control for auto-oxidation of the L-DOPA substrate. No L-DOPA oxidation was identified in the hemocytes of another fresh water snail, *Lymnaea stagnalis* [36]. In our study, we detected very low laccase activity in the hemocytes of *Biomphalaria.* This may be due to the high sensitivity of our assay, allowing detection of residual activity from the cells’ membrane, as hypothesized in the oyster *C. virginica* [44]. These results demonstrate the importance of testing both cellular (hemocytes) and acellular (plasma) fractions in order to accurately localize the PO activity.

In other arthropods such as insects or crustaceans, PO activity is mainly localized in the hemocytes and is typically tyrosinase activity, using tyrosine present in the hemolymph [16, 56]. The differences in the type of PO activity (tyrosinase vs laccase) and the enzyme localization (hemocyte vs plasma) in arthropods and mollusks raise intriguing questions about the evolution of PO enzymes in these two phyla.

Addition of trypsin, a serine protease enzyme, demonstrated the presence of circulating prolaccase-like enzyme in the snail hemolymph. One possible alternative explanation, that trypsin makes the active site of the laccase-like enzyme more accessible [57], is unlikely because trypsin had no impact on laccase activity of infected snails (7-9 weeks). Prolaccase-like enzymes may be activated by endogenous serine proteases produced by the snail [38] or by exogenous serine proteases produced by pathogens [17, 58]. Non-activated PO enzymes are common in all PO systems characterized [56]. In arthropods, comparisons of intrinsic PO activity (PO enzymes activated by endogenous proteases) and the total PO activity (intrinsic PO + PO enzymes activated by exogenous proteases) provide an important parameter used to assess immunocompetence and health status in an ecological context [17].

### Impact of *S. mansoni* on *B. glabrata* PO activity

We examined the impact of *S. mansoni* infection on *B. glabrata* laccase activity over 9 weeks. Our results demonstrate a strong reduction in laccase activity in the snail hemolymph starting 7 weeks post-infection. Similar negative impacts of parasitic infection on PO activity were found in the oyster *C. virginica* and the mussel *Geukensia demissa* infected by *Perkinsus marinus* [44], while PO activity of the abalone *H. diversicolor* was reduced by infection with *Vibrio parahaemolyticus* [59]. Susceptibility to QX disease (caused by the paramyxean protozoan *Marteilia sydneyi* [60, 61]) in the Sydney rock oyster *Saccostrea glomerata* is also associated with a rapid decrease in PO activity [62]. During the late stage of infection (7-9 weeks) trypsin had no effect on laccase activity. We interpret this result to mean that that total PO activity drops to the same level as intrinsic PO activity in infected snails, because laccase-like enzyme production has collapsed.

The decrease in PO activity in the snail hemolymph is correlated with the development of the *S. mansoni* secondary sporocysts in these experiments. This intramolluscan parasite stage metabolizes snail tissues, such as the hepatopancreas and the albumen gland, organs that are involved in the protein production [63], potentially including the laccase-like enzyme. However, parasite growth does not have any negative impact on snail PO activity during the first 6 weeks. These results are consistent to those obtained by Seppälä and Leicht [37] in the freshwater snail *Lymnaea stagnalis.* They observed no change in PO activity after the injection of trematode (*Plagiorchis sp*.) infected gonads compared to the injection of uninfected gonad. In contrast, injection of bacteria (*E.coli* or *Micrococcus lysodeikticus)* led to a decrease in PO activity.

## Conclusion

We report and characterize laccase activity in the hemolymph of *B. glabrata* and *B. alexandrina*, intermediate hosts of the human blood fluke *S. mansoni*. Based on these findings, we employed a sensitive and specific spectrophotometric assay using PPD as substrate. This paves the way to better understanding of the role of PO enzymes in molluscan biology and host defense. Infection of snails with *S. mansoni* had a severe impact on PO production, but only after 6 weeks of infection. This strong negative effect of the parasite may be explained by the fact that secondary sporocysts of *S. mansoni* metabolize snail tissues involved in protein production, during their development and cercariae production.

## Additional file

Additional file 1: Figure S1. Absorption spectrum of the product of PPD oxidation by the laccase-like enzyme in the hemolymph of *Biomphalaria spp.* The absorption spectrum was determined using continuous wavelength scanning from 375 to 675 nm. The maximum absorption, after 2 h of reaction with 50 mM of PPD, corresponds to wavelength of 465 nm. (PDF 4 kb)
