# Supplementary figures and images for: Characterization of hemolymph phenoloxidase activity in two Biomphalaria snail species and impact of Schistosoma mansoni infection
Source: Parasit Vectors. 2016 Jan 22;9:32. doi: 10.1186/s13071-016-1319-6 (PMC4722754; doi:10.1186/s13071-016-1319-6)

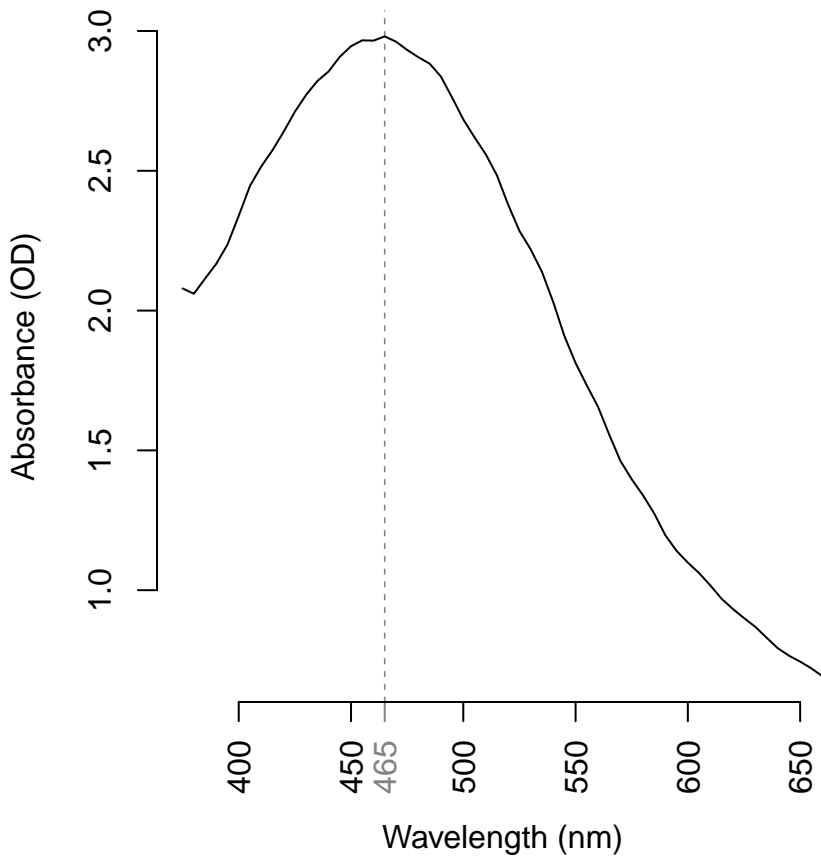

Supplement: Additional file 1: Figure S1. — Absorption spectrum of the product of PPD oxidation by the laccase-like enzyme in the hemolymph of Biomphalaria spp. The absorption spectrum was determined using continuous wavelength scanning from 375 to 675 nm. The maximum absorption, after 2 h of reaction with 50 mM of PPD, corresponds to wavelength of 465 nm. (PDF 4 kb) [file 13071_2016_1319_MOESM1_ESM.pdf]
